# Meat Quality and Fatty Acid Profiles of Chinese Ningxiang Pigs Following Supplementation with *N*-Carbamylglutamate

**DOI:** 10.3390/ani10010088

**Published:** 2020-01-06

**Authors:** Yueteng Xing, Xin Wu, Chunyan Xie, Dingfu Xiao, Bin Zhang

**Affiliations:** 1 Hunan Co-Innovation Center of Safety Animal Production, College of Animal Science and Technology, Hunan Agricultural University, Changsha 410128, China; xingyuet@126.com (Y.X.); xiechunyan@hunau.edu.cn (C.X.); xiaodingfu2001@163.com (D.X.); 2 Key Laboratory of Agro-Ecological Processes in Subtropical Region, Institute of Subtropical Agriculture, Chinese Academy of Sciences, Changsha 410125, China; 3 Institute of Biological Resources, Jiangxi Academy of Sciences, Nanchang 330096, China

**Keywords:** *N*-carbamylglutamate, meat quality, fatty acid, lipid metabolism, Ningxiang pigs

## Abstract

**Simple Summary:**

*N*-carbamylglutamate (NCG) has been demonstrated to promote the synthesis of endogenous arginine and improve reproductive performance. In the present study, we found that dietary NCG supplementation improved meat quality of a Chinese fat-type pig by increasing muscle tenderness and Phe concentration, and optimizing fatty acid profiles in different tissues. These results provided scientific evidence for the application of NCG as a feed additive in finishing pigs.

**Abstract:**

The present study evaluated the effects of dietary *N*-carbamylglutamate (NCG) on carcass traits, meat quality, and fatty acid profiles in the longissimus dorsi muscle and adipose tissues of Chinese Ningxiang pigs. A total of 36 castrated female pigs with a similar initial weight (43.21 ± 0.57 kg) were randomly assigned to two treatments (with six pens per treatment and three pigs per pen) and fed either a basal diet or a basal diet supplemented with 0.08% NCG for 56 days. Results showed that dietary NCG reduced shear force (*p* = 0.004) and increased drip loss (*p* = 0.044) in longissimus dorsi muscle of Ningxiang pigs. Moreover, increased levels of oleic acid (C18:1n9c) (*p* = 0.009), paullinic acid (C20:1) (*p* = 0.004), and α-linolenic acid (C18:3n3) (*p* < 0.001), while significant reduction in the proportions of arachidonic acid (C20:4n6) (*p* < 0.001) and polyunsaturated fatty acid (PUFA) (*p* = 0.017) were observed in the longissimus dorsi muscle of pigs fed NCG when compared with those fed the control diet. As for adipose tissues, the C20:1 (*p* = 0.045) proportion in dorsal subcutaneous adipose (DSA), as well as the stearic acid (C18:0) (*p* = 0.018) level in perirenal adipose (PA) were decreased when pigs were fed the NCG diet compared with those of the control diet. In contrast, the margaric acid (C17:0) (*p* = 0.043) proportion in PA were increased. Moreover, the NCG diet produced PA with a greater proportion of total PUFAs (*p* = 0.001) (particularly linoleic acid (C18:2n6c) (*p* = 0.001)) compared with those produced by the control diet. These findings suggest that dietary NCG has beneficial effects by decreasing the shear force and improving the healthfulness of fatty acid profiles, providing a novel strategy for enhancing meat quality of pigs.

## 1. Introduction

For the pork industry, fat and fatty acids (whether in muscle or adipose tissues) contribute importantly to various aspects of meat quality (e.g., flavor, taste) and are central to nutritional value [1]. However, fat-type pigs exhibiting excessive amounts of subcutaneous adipose tissues have been recognized as detrimental to carcass quality [2]. Moreover, imbalanced fatty acid composition is even harmful to the consumer [3]. Therefore, increasing attention is mainly focused on safer, healthier, and taster of meat. Indeed, the use of nutritional approaches to optimize meat fatty acid composition has been a popular research topic, for example, supplying specific additives in diets such as linseeds, plant extracts [4,5]. The application of *N*-carbamylglutamate (NCG) as a dietary supplement for the health of humans and animals also has gained increasing interest [6,7,8].

NCG, as an effective and metabolically stable analog of *N*-acetylglutamate, promotes the synthesis of endogenous arginine [9,10]. What’s more, NCG facilitates muscle protein synthesis [11], protects the small intestinal morphologic [12] and improves reproductive performance [13,14]. Moreover, new evidence indicated that NCG can enhance the antioxidant capability in the plasma, spleen, liver, and jejunum of rats [12,15,16]. Our previous study also proved that NCG is a non-toxic substance with no genotoxicity in rats [17]. However, few studies regard the effect of NCG on meat quality of pigs, and whether fatty acid metabolism may be involved in the regulation of the process. Advances in seabass demonstrated that NCG alleviates liver metabolic disease and hepatic inflammation via inhibiting ERK1/2-mTOR-S6K1 signaling pathway, and the ameliorated function is closely associated with the improved lipid metabolism indices, for example, lower plasma very low-density lipoprotein and hepatic triglyceride and non-esterified fatty acid accumulation [10]. Ningxiang pig, as one of Chinese indigenous fat-type breeds, exhibits early sexual maturity, tender succulent flavor, strong adaptability and resistance, plays an increasingly significant role in the pork industry [18]. Given the foregoing, we hypothesized that dietary NCG may affect meat quality traits of Ningxiang pigs through influencing lipid metabolism. Therefore, the purpose of the present study was to evaluate the effects of dietary NCG on carcass traits, meat quality, and fatty acid profiles in different tissues of Ningxiang pigs.

## 2. Materials and Methods

### 2.1. Ethics Statement

Animal experiments were approved and performed following regulations and guidelines established by the Animal Welfare Committee of Hunan Agricultural University (Changsha, China) (No. 2013-06). 

### 2.2. Animals and Experimental Design

Thirty-six castrated female Chinese Ningxiang pigs with a similar initial weight (43.21 (_SEM_ 0.57) kg) were selected from the same herd. Pigs were randomly allocated to two treatment groups with six pens per treatment and three pigs per pen. Pigs were fed a basal diet, unsupplemented (Control group) or supplemented with 0.08% NCG (NCG group) for a 56 day period. NCG, 98.30% purity, supplied by Changsha Green Top Biotech Co., Ltd. (Changsha, China), and the dose of which was based on the previous study with pigs [19]. The ingredient composition and nutrient content of the basal diet (meets recommendations of Chinese National Feeding Standard for Swine (2004)) are shown in Table 1. Feed and water were provided ad libitum throughout the experiment period. The feeding experiment was carried out in Hunan Liushahe Spotted Pig Eco-Farm Co., Ltd. (Changsha, China). 

At the end of the experiment (pigs with final body weight of 74.13 (_SEM_ 1.34) kg), one pig with medium weight per pen was chosen and slaughtered by exsanguination after electrical stunning. Samples of the longissimus dorsi muscle and adipose tissues were immediately resected from the right side of the carcass, and flash-frozen using liquid N_2_, then stored at −20 °C for determination of the chemical composition.

### 2.3. Carcass Trait and Meat Quality Measurements

At slaughter, carcass weight was recorded after evisceration so that carcass yields could be calculated. The other carcass traits (obtained from the left side of the carcass) including carcass length, loin muscle area, average backfat thickness of first- and last-rib and last-lumbar vertebra were measured by the previous methods [20].

The pH values of the longissimus dorsi muscle were measured at 45 min (pH_45min_) and 24 h (pH_24h_) postmortem, using a portable pH meter (pH-STAR, SFK-Technology, Denmark). The longissimus dorsi muscle colors were assessed objectively in triplicate, from a freshly cut surface with the parameters L* (brightness), a* (redness), and b* (yellowness) at 45 min postmortem, using a hand-held color meter (CR 300, Minolta Co. Ltd., Osaka, Japan). Shear force and drip loss of the longissimus dorsi muscle were measured according to previously reported methods [21]. 

### 2.4. Hydrolytic Amino Acid Analysis

Muscle amino acid contents were determined by an ion-exchange amino acid analyzer (Hitachi L-8900, Tokyo, Japan). Briefly, about 0.1 g of ground freeze-dried sample of the longissimus dorsi muscle was hydrolyzed in 10 mL of 6 mol/L HCl for 24 h at 110 °C. The solution was then adjusted to a volume of 100 mL and 1 mL of the settled solution was filtered through a 0.45 μm membrane and then the filtered solution after 10-fold dilution was used for amino acid analysis [22].

### 2.5. Fatty Acid Composition Analysis

To determine the fatty acid composition, lipid extraction and transesterification were performed according to previously reported procedures [23]. Briefly, the thawed longissimus dorsi muscle or adipose tissue section was blended with chloroform-methanol (1:1, v/v) containing butylated hydroxytoluene and was homogenized. Fatty acid methyl esters were analyzed using gas chromatography (Agilent 6890N equipped with a flame ionization detector and a CP-Sil 88 fused silica open tubular capillary column). The initial oven temperature was set at 45 °C for 4 min, and then raised to 175 °C at 13 °C/min, held at 175 °C for 27 min and then increased to 215 °C at 4 °C/min and then held at 215 °C for 35 min. The injector and detector temperatures were set at 250 °C. The carrier gas was hydrogen at a flow rate of 30 mL/min. Fatty acids were identified through comparisons to the retention time of standard esters, and the concentration of individual fatty acid was quantified according to the peak area and expressed as a percentage of the total area [24].

### 2.6. Statistical Analysis

Data were analyzed using unpaired, two-tailed Student’s *t*-test of SPSS 17.0 (2012, SPSS Inc., Chicago, IL, USA). Values were expressed as mean ± standard error of mean (SEM), and *p-*values < 0.05 were considered statistically significant. 

## 3. Results

Table 2 presents that NCG has no significant differences in carcass traits including slaughter yield, straight/oblique length and loin muscle area. In addition, pH values and muscle colors (i.e., L*, a*, and b*) were not affected by any of the dietary treatments (*p* > 0.05). However, drip loss was significantly increased by 30.32% (*p* = 0.044), while shear force was significantly decreased by 29.51% (*p* = 0.004) after NCG supplementation.

The effects of NCG supplementation on amino acid profiles in the longissimus dorsi muscle of Ningxiang pigs are listed in Table 3. NCG tended to increase the concentration of phenylalanine (Phe) (*p* = 0.066) in the longissimus dorsi muscle. Moreover, increased levels of oleic acid (C18:1n9c) (*p* = 0.009), paullinic acid (C20:1) (*p* = 0.004), α-linolenic acid (C18:3n3) (*p* < 0.001) and docosahexaenoic acid (C22:6n3) (*p* = 0.082), while significant reduction in the proportions of arachidonic acid (C20:4n6) (*p* < 0.001) and polyunsaturated fatty acid (PUFA) (*p* = 0.017) were observed in the longissimus dorsi muscle of pigs fed NCG when compared with pigs fed the control diet (Table 4).

As for adipose tissues, Table 5, Table 6 and Table 7 present the effects of NCG supplementation on the fatty acid profiles in dorsal subcutaneous adipose (DSA), abdominal subcutaneous adipose (ASA), and perirenal adipose (PA) respectively. The C20:1 (*p* = 0.045) proportion in DSA and C20:4n6 (*p* = 0.070) in ASA, as well as the stearic acid (C18:0) (*p* = 0.018) and C20:1 (*p* = 0.063) levels in PA decreased in the pigs that were fed the NCG diet compared with those of the control diet. In contrast, the margaric acid (C17:0) (*p* = 0.043) and C18:3n3 (*p* = 0.071) proportions in PA were increased. Moreover, the NCG diet produced these adipose tissues with a greater proportion of total PUFAs (*p* < 0.1) (particularly linoleic acid (C18:2n6c) (*p* < 0.1)) compared with those produced by the control diet.

## 4. Discussion

New research shows that NCG may improve lipid metabolism with decreased plasma very low-density lipoprotein, hepatic triglyceride and non-esterified fatty acid accumulation, down-regulated fatty acid and cholesterol synthesis, and simultaneously increased lipolysis gene mRNA levels of fish [10]. However, few studies regard the effect of NCG on meat quality of pigs. The present study for the first time reported the use of NCG as a feed additive for Chinese local pigs, to determine whether it could impact or even improve fatty acid profiles in different tissues. Fatty acids are essential components of membrane phospholipids, and many of them have been associated with cardiovascular, metabolic and neuropsychiatric disorders [3].

No significant differences were observed in carcass traits of Ningxiang pigs between groups under the conditions of our study, which contradicted previous findings that NCG is effective to increase longissimus dorsi muscle area and decrease back fat accretion [25]. The possible reasons for this discrepancy could be attributed to the diet factors (regular vs. reduced protein level) or the type of pigs (fat genotype vs. lean phenotype) used in studies. As mentioned previously, there is increasing interest in meat quality for consumers, particularly in tenderness and juiciness [3]. Among them, tenderness is critically important from a sensory viewpoint. In the present study, pork from the NCG diet had a lower shear force value than from the control diet, indicating a more tender texture. Thus, NCG supplementation in the swine diet may be a good nutritional strategy for tender pork production. Drip loss, another quality measure of pork, is a natural phenomenon encountered during refrigerated storage of fresh meat. Generally, meat with a high drip loss percentage would lead to unattractive appearance and low consumer acceptance, which eventually reduce economic benefits [26]. Another major finding from the present study was that dietary NCG had a significant adverse influence on drip loss compared to the control group. The moisture retention potential of fresh pork muscle is ostensibly related to some specific fatty acidss [27]. It appears that total saturated fatty acids (SFAs) may be negatively associated with drip loss, suggesting that decreased SFA in the longissimus dorsi muscle may be an influencing factor for drip loss. The precise mechanism underlying this effect currently requires further investigations.

Muscle is the largest reservoir of amino acids in the body, and essential amino acids in meat can offer high nutritional values [24,28]. In addition, amino acid composition determines the flavor of meat, which is also an important source of essential amino acids in human diets [29]. Recent research demonstrated NCG promotes intestinal absorption and transport of amino acids or peptides in suckling lambs via regulating the mTOR signaling pathway [30]. Indeed, NCG could increase protein synthesis in skeletal muscle [11]. In the present study, NCG increased the concentration of Phe in the longissimus dorsi muscle slightly, which was consistent with the previous result obtained by Liu et al. (2016) that the Phe content is increased by NCG intake in rat plasma [31]. Phe is an essential amino acid for humans, and of great relevance to assessing the nutritional value of meat. One study reports that NCG could significantly decrease homogentisate, an intermediate of the metabolic breakdown of Phe [32]. Besides, the coordinated activity of certain amino acid transporters in the cellular membranes may partially response to the intracellular presence of available amino acids [33]. These transporters can sense the availability of amino acids, relay nutrient signals to the cell interior, move amino acid in or out of the cells, and launch a series of cascade responses, thus exhibiting a dual transporter and receptor function [34]. A study by Yang et al. (2013) found that NCG ameliorates the absorptive capacity of weaned piglets by increasing mRNA expression of Slc6a19, Slc7a9 and protein abundance of ASCT2, B^0^AT1 and b^0,+^AT in the jejunum. These altered transporters involved in mediating the transfer of Phe may contribute to Phe increment in the longissimus dorsi muscle of Ningxiang pigs [35].

A certain amount of fat in pork meat is favorably related to the palatability of the juiciness, odor, and flavor of pork meat when it is cooked as a roast or chop. Accordingly, the fatty acid composition of muscle seals the nutritional quality of pork, for example, PUFA content is positively correlated with meat off-flavor [36,37]. Various studies have demonstrated that NCG supplementation could affect lipid and energy metabolism (such as acetoacetate, acetone, lactate, creatine) in rats [31,32]. These results indicate that NCG may have beneficial effects on the taste and tenderness of pork since these meat characteristics are closely related to fatty acid composition [1]. In the present study, the percentage of each fatty acid respect to all fatty acids within the fraction was calculated, and the NCG diet produced the longissimus dorsi muscle with a greater concentrations of C18:1n9c, C20:1, C18:3n3, and C22:6n3, and with a lower level of total PUFAs (particularly pro-inflammatory factor C20:4n6) compared with those produced by the control diet, indicated the reassignment of these fatty acids. These findings are partly consistent with the previous result obtained by Ye et al. (2017) that the muscular C20:4n6 content is decreased by NCG intake in finishing pigs fed the reduced protein diet [25]. The possible reasons could be attributed that NCG could increase endogenous NO production, which accelerates the synthesis of eicosanoids, and results in the C20:4n6 proportion decrease [19]. C18:1n9c is the most abundant showing levels of 90% total monounsaturated fatty acids (MUFAs) and positively correlated with flavor, and also described as a regulator of immune function and cholesterol levels [38,39]; whereas C20:4n6 is capable of being converted into numerous inflammatory mediators and stimulating the pathogenesis through the prostacyclin pathway [40,41]. Notably, the percentage of C18:3n3 and C22:6n3 were increased in the present study. C18:3n3 and C22:6n3 are both types of n-3 series fatty acids and have been well studied for their roles in reducing the risk factors of disordered lipid metabolism, suggesting that these changes may be beneficial in inhibiting fat accumulation [42].

The effect of NCG on fatty acid composition was only evaluated in the longissimus muscle [25], limited information is available on the fatty acid composition of adipose tissues. Indeed, lipid synthesis mainly occurs in adipose tissues of pigs. Subcutaneous and visceral adipose tissues with different anatomical locations show specific development and deposition, especially in de novo synthesized fatty acids due to desaturation and elongation [43,44]. NCG is involved in regulating the metabolism of energy substrates through nitric oxide production [19,45]. Nitric oxide, as a signaling molecule, stimulates glucose and fatty acid oxidation, enhances lipolysis, and inhibits lipogenesis in subcutaneous adipose tissues [46,47]. It seems that tissue-specific manner of dietary NCG on fatty acid composition in adipose tissues of Ningxiang pigs exists. Our results showed NCG supplementation resulted in an increased amount of C18:2n6c in these adipose tissues, which mainly explained the higher PUFA percentage. This shift towards greater unsaturation in adipose tissues and an increase in C18:2n6c could lead to stimulating lipid oxidation of the pork fat, and have a hypocholesterolemic effect and thereby slow the development of atherosclerosis for the consumer [48]. However, such depot fats exhibiting a high content in C18:2n6c are often soft with a decrease in their storage capacity and their technological quality, C18:2n6c also elongates and desaturates to form C20:4n6 in the body, a precursor to pro-inflammatory compound that can have detrimental effects on health [49]. Given the complexity of the nutritional role of linoleic acid, an appropriate level of intake should be considered [50]. It is interesting to note that the decrease in C18:0 proportion was of greater magnitude in PA than in subcutaneous fat, and the opposite occurred for C16:0, thus indicating different regulatory effects of NCG. Besides, the percentage of C20:1 in DSA and PA was decreased, but further investigations into the potential mechanism of NCG on fatty acid metabolism are, therefore, warranted. Consequently, feeding NCG may be useful in modifying pork fatty acid composition to meet market demands (i.e., for either lower SFA and specific MUFA, or increased PUFA).

## 5. Conclusions

Dietary NCG did exert beneficial effects on pork quality by decreasing shear force in the longissimus dorsi muscle, as well as improving fatty acid profiles (C18:1n9c, C18:3n3, C20:4n6 in muscle, and C18:2n6c in adipose tissues were accentuated respectively) in a tissue-specific manner, but with adverse impact on drip loss of Ningxiang pigs. In short, results from this study indicate that NCG is feasible as a feed additive for fat-type pigs to improve meat quality and fatty acid composition, but whether the similar effects of NCG in lean phenotype pigs or not still requires more investigations.

## Figures and Tables

**Table 1:** Composition and nutrient levels of the basal diet (air-dry basis).

Ingredient %		Nutrient Content ^1^ %	
Corn	65.50	DE (MJ/kg)	12.49
Soybean meal	6.50	Crude protein	11.91
Wheat bran	24.00	Crude ash	4.94
Limestone powder	1.20	Ether extract	3.35
Zeolite powder	0.64	Calcium	0.66
Rice bran	0.40	Total phosphorus	0.50
Calcium hydrogen phosphate	0.60	Available phosphorus	0.25
l*-*lysine-HCl (70%)	0.50	SID lysine	0.64
l-threonine (98.5%)	0.05	SID threonine	0.38
Salt	0.36	SID methionine	0.18
Vitamin and mineral premixs ^2^	0.25	SID methionine + cysteine	0.37

^1^ DE (digestible energy) is a calculated value and the others are measured values. ^2^ The vitamin and mineral premixs provide the following per kg of the diet: retinol 3000 IU, cholecalciferol 400 IU, vitamin E 28.0 IU, vitamin K_3_ 2.0 mg, thiamine 3.6 mg, riboflavin 7.0 mg, pyridoxine 2.1 mg, cyanocobalamin 16.0 μg, folic acid 0.4 mg, pantothenic acid 12.0 mg, copper (as copper sulfate) 4.0 mg, selenium (as sodium selenite) 0.2 mg, zinc (as zinc sulfate) 62.0 mg, iron (as iron sulfate) 76.8 mg, manganese (as manganese sulfate) 8.44 mg.

**Table 2:** Effects of *N*-carbamylglutamate on carcass and meat quality traits in Ningxiang pigs.

Item	Control	NCG	*p*-Value
Carcass weight kg	56.52 *±* 0.31	56.10 *±* 1.11	0.730
Slaughter yield %	73.60 *±* 0.73	73.68 *±* 0.47	0.926
Straight length cm	80.67 *±* 1.61	80.33 *±* 0.84	0.858
Oblique length cm	71.83 *±* 1.17	71.17 *±* 0.70	0.635
Average backfat thickness mm	45.75 *±* 1.58	45.08 *±* 1.50	0.766
Loin muscle area cm^2^	17.89 *±* 0.56	18.65 *±* 1.00	0.521
pH_45min_	6.63 *±* 0.09	6.67 *±* 0.07	0.770
pH_24h_	5.80 *±* 0.09	5.92 *±* 0.07	0.285
Drip loss %	1.55 *±* 0.18	2.02 *±* 0.09	0.044
Shear force kg	7.93 *±* 0.44	5.59 *±* 0.45	0.004
Color			
Lightness (L*)	43.20 *±* 0.61	43.19 *±* 0.67	0.989
Redness (a*)	7.26 *±* 0.53	7.13 *±* 0.18	0.814
Yellowness (b*)	3.11 *±* 0.16	2.98 *±* 0.06	0.474

Values are presented as means ± SEM, *n* = 6.

**Table 3:** Effect of *N*-carbamylglutamate on hydrolytic amino acid concentration in the longissimus dorsi muscle of Ningxiang pigs, g/100 g.

Amino Acid	Control	NCG	*p*-Value
Asp	7.31 *±* 0.10	7.50 *±* 0.11	0.228
Thr	4.46 *±* 0.07	4.56 *±* 0.06	0.325
Ser	3.82 *±* 0.05	3.90 *±* 0.02	0.136
Glu	13.41 *±* 0.15	13.66 *±* 0.05	0.168
Gly	3.53 *±* 0.05	3.60 *±* 0.02	0.206
Ala	5.07 *±* 0.07	5.10 *±* 0.01	0.616
Cys	0.90 *±* 0.03	0.91 *±* 0.02	0.663
Val	4.50 *±* 0.05	4.57 *±* 0.03	0.254
Met	2.23 *±* 0.04	2.13 *±* 0.07	0.250
Ile	4.14 *±* 0.04	4.20 *±* 0.02	0.225
Leu	7.30 *±* 0.05	7.45 *±* 0.01	0.124
Tyr	2.73 *±* 0.04	2.76 *±* 0.02	0.555
Phe	3.64 *±* 0.01	3.73 *±* 0.01	0.066
Lys	7.90 *±* 0.09	8.08 *±* 0.02	0.118
His	4.12 *±* 0.08	4.29 *±* 0.07	0.114
Arg	5.52 *±* 0.06	5.63 *±* 0.01	0.130

Values are presented as means ± SEM, *n* = 6.

**Table 4:** Effect of dietary *N-*carbamylglutamate on long-chain fatty acid composition (% of total fatty acids) in the longissimus dorsi muscle of Ningxiang pigs.

Long-Chain Fatty Acid	Control	NCG	*p*-Value
Myristic (C14:0)	1.43 *±* 0.41	1.48 *±* 0.78	0.595
Palmitic acid (C16:0)	28.31 *±* 1.89	27.99 *±* 0.28	0.374
Margaric acid (C17:0)	0.23 *±* 0.01	0.25 *±* 0.01	0.352
Stearic acid (C18:0)	13.90 *±* 0.22	13.79 *±* 0.31	0.777
Arachidic acid (C20:0)	0.21 *±* 0.01	0.21 *±* 0.01	0.799
Palmitoleic acid (C16:1)	3.97 *±* 0.08	4.11 *±* 0.26	0.617
Elaidic acid (C18:1n9t)	0.19 *±* 0.01	0.20 *±* 0.01	0.401
Oleic acid (C18:1n9c)	38.69 *±* 0.57	40.96 *±* 0.40	0.009
Paullinic acid (C20:1)	0.25 *±* 0.01	0.29 *±* 0.01	0.004
Linoleic acid (C18:2n6c)	9.93 *±* 0.32	10.13 *±* 0.53	0.759
α-Linolenic acid (C18:3n3)	0.08 *±* 0.00	0.16 *±* 0.01	<0.001
Dihomo-γ-linolenic acid (C20:3n6)	0.33 *±* 0.03	0.42 *±* 0.05	0.159
Arachidonic acid (C20:4n6)	2.29 *±* 0.75	0.42 *±* 0.05	<0.001
Docosahexaenoic acid (C22:6n3)	0.14 *±* 0.01	0.19 *±* 0.02	0.082
ΣSFA	44.08 *±* 0.39	43.72 *±* 0.37	0.515
ΣMUFA	43.10 *±* 0.63	44.73 *±* 1.16	0.246
ΣPUFA	12.82 *±* 0.39	10.64 *±* 0.66	0.017

ΣSFA = sum of saturated fatty acids; ΣMUFA = sum of monounsaturated fatty acids; ΣPUFA = sum of polyunsaturated fatty acids; Values are presented as means ± SEM, *n* = 6.

**Table 5:** Effect of dietary *N*-carbamylglutamate on long-chain fatty acid composition (% of total fatty acids) in the dorsal subcutaneous adipose of Ningxiang pigs.

Long-Chain Fatty Acid	Control	NCG	*p*-Value
Myristic (C14:0)	1.23 *±* 0.03	1.22 *±* 0.03	0.806
Palmitic acid (C16:0)	24.02 *±* 0.29	23.73 *±* 0.27	0.566
Margaric acid (C17:0)	0.19 *±* 0.01	0.20 *±* 0.01	0.344
Stearic acid (C18:0)	15.38 *±* 0.39	15.23 *±* 0.50	0.815
Arachidic acid (C20:0)	0.26 *±* 0.02	0.24 *±* 0.02	0.393
Palmitoleic acid (C16:1)	1.49 *±* 0.04	1.54 *±* 0.06	0.570
Elaidic acid (C18:1n9t)	0.10 *±* 0.01	0.10 *±* 0.01	0.834
Oleic acid (C18:1n9c)	47.44 *±* 0.63	47.79 *±* 0.72	0.718
Paullinic acid (C20:1)	1.22 *±* 0.05	1.03 *±* 0.06	0.045
Linoleic acid (C18:2n6c)	7.79 *±* 0.21	8.49 *±* 0.25	0.061
α-Linolenic acid (C18:3n3)	0.33 *±* 0.01	0.35 *±* 0.01	0.181
Dihomo-γ-linolenic acid (C20:3n6)	0.09 *±* 0.01	0.10 *±* 0.01	0.726
Arachidonic acid (C20:4n6)	0.13 *±* 0.01	0.12 *±* 0.00	0.165
Docosahexaenoic acid (C22:6n3)	0.05 *±* 0.00	0.05 *±* 0.00	0.922
ΣSFA	41.23 *±* 0.65	40.18 *±* 0.95	0.381
ΣMUFA	50.34 *±* 0.68	50.67 *±* 0.81	0.764
ΣPUFA	8.43 *±* 0.22	9.15 *±* 0.27	0.061

ΣSFA = sum of saturated fatty acids; ΣMUFA = sum of monounsaturated fatty acids; ΣPUFA = sum of polyunsaturated fatty acids; Values are presented as means ± SEM, *n* = 6.

**Table 6:** Effect of dietary *N*-carbamylglutamate on long-chain fatty acid composition (% of total fatty acids) in the abdominal subcutaneous adipose of Ningxiang pigs.

Long-Chain Fatty Acid	Control	NCG	*p*-Value
Myristic (C14:0)	1.52 *±* 0.05	1.51 *±* 0.05	0.878
Palmitic acid (C16:0)	23.88 *±* 0.16	23.51 *±* 0.18	0.155
Margaric acid (C17:0)	0.21 *±* 0.02	0.22 *±* 0.00	0.819
Stearic acid (C18:0)	12.15 *±* 0.41	11.77 *±* 0.47	0.558
Arachidic acid (C20:0)	0.18 *±* 0.01	0.16 *±* 0.01	0.148
Palmitoleic acid (C16:1)	2.31 *±* 0.05	2.32 *±* 0.15	0.985
Elaidic acid (C18:1n9t)	0.10 *±* 0.01	0.10 *±* 0.01	0.511
Oleic acid (C18:1n9c)	50.01 *±* 0.55	50.21 *±* 0.67	0.827
Paullinic acid (C20:1)	1.00 *±* 0.08	0.87 *±* 0.08	0.275
Linoleic acid (C18:2n6c)	7.90 *±* 0.23	8.63 *±* 0.29	0.076
α-Linolenic acid (C18:3n3)	0.36 *±* 0.01	0.36 *±* 0.01	0.889
Dihomo-γ-linolenic acid (C20:3n6)	0.10 *±* 0.01	0.12 *±* 0.02	0.337
Arachidonic acid (C20:4n6)	0.17 *±* 0.01	0.14 *±* 0.00	0.070
Docosahexaenoic acid (C22:6n3)	0.06 *±* 0.00	0.06 *±* 0.00	0.996
ΣSFA	37.95 *±* 0.47	37.17 *±* 0.65	0.354
ΣMUFA	53.43 *±* 0.68	53.49 *±* 0.72	0.958
ΣPUFA	8.62 *±* 0.24	9.35 *±* 0.31	0.093

ΣSFA = sum of saturated fatty acids; ΣMUFA = sum of monounsaturated fatty acids; ΣPUFA = sum of polyunsaturated fatty acids; Values are presented as means ± SEM, *n* = 6.

**Table 7:** Effect of dietary *N*-carbamylglutamate on long-chain fatty acid composition (% of total fatty acids) in the perirenal adipose of Ningxiang pigs.

Long-Chain Fatty Acid	Control	NCG	*p*-Value
Myristic (C14:0)	1.36 *±* 0.04	1.44 *±* 0.06	0.331
Palmitic acid (C16:0)	24.20 *±* 0.47	24.21 *±* 0.51	0.992
Margaric acid (C17:0)	0.21 *±* 0.01	0.25 *±* 0.01	0.043
Stearic acid (C18:0)	18.26 *±* 0.44	16.74 *±* 0.29	0.018
Arachidic acid (C20:0)	0.24 *±* 0.03	0.20 *±* 0.01	0.244
Palmitoleic acid (C16:1)	1.23 *±* 0.10	1.45 *±* 0.10	0.159
Elaidic acid (C18:1n9t)	0.11 *±* 0.01	0.11 *±* 0.00	0.499
Oleic acid (C18:1n9c)	44.94 *±* 0.89	45.03 *±* 0.76	0.940
Paullinic acid (C20:1)	0.98 *±* 0.10	0.73 *±* 0.06	0.063
Linoleic acid (C18:2n6c)	7.77 *±* 0.29	9.14 *±* 0.07	0.001
α-Linolenic acid (C18:3n3)	0.33 *±* 0.01	0.36 *±* 0.00	0.071
Dihomo-γ-linolenic acid (C20:3n6)	0.09 *±* 0.01	0.09 *±* 0.02	0.923
Arachidonic acid (C20:4n6)	0.15 *±* 0.01	0.15 *±* 0.01	0.642
Docosahexaenoic acid (C22:6n3)	0.06 *±* 0.00	0.07 *±* 0.00	0.618
ΣSFA	44.27 *±* 0.79	42.84 *±* 0.66	0.193
ΣMUFA	47.27 *±* 0.95	47.32 *±* 0.70	0.968
ΣPUFA	8.46 *±* 0.31	9.84 *±* 0.07	0.001

ΣSFA = sum of saturated fatty acids; ΣMUFA = sum of monounsaturated fatty acids; ΣPUFA = sum of polyunsaturated fatty acids; Values are presented as means ± SEM, *n* = 6.
